# T and B lymphocytes in the brains of dogs with concomitant seropositivity to three pathogenic protozoans: *Leishmania chagasi*, *Toxoplasma gondii* and *Neospora caninum*

**DOI:** 10.1186/1756-0500-6-226

**Published:** 2013-06-08

**Authors:** Keila Priscilla Sakamoto, Guilherme Dias de Melo, Gisele Fabrino Machado

**Affiliations:** 1 Laboratory of Applied Pathology (LAPAP), College of Veterinary Medicine, UNESP – Univ Estadual Paulista, Rua Clóvis Pestana, 739, CEP 16050-680, Araçatuba, São Paulo, Brazil

**Keywords:** CD3, CD79α, Central Nervous System, Inflammation, Lymphocyte, Neosporosis, Toxoplasmosis, Visceral Leishmaniasis

## Abstract

**Background:**

Visceral leishmaniasis is a disease with great variability regarding the clinical manifestations in humans and dogs. Chronically infected dogs may develop neurological disorders, however, there are few reports that characterize the lesions and make clear the pathogenesis of the canine cerebral leishmaniasis. Concomitant with *Leishmania chagasi*, dogs may be infected by opportunistic pathogens, such as *Toxoplasma gondii* and *Neospora caninum*, which may contribute to the occurrence of lesions in the central nervous system. Hence, we aimed to compare the T and B lymphocytes population in the brains of infected dogs with seropositivity to *L. chagasi, T. gondii* and *N. caninum* concurrently (n = 24), seropositivity only to *L. chagasi* (n = 31), and seropositivity to *T. gondii* and *N. caninum* (n = 16). Uninfected dogs were used as control (n = 10).

**Results:**

Inflammatory lesions, characterised by mononuclear cell accumulation, composed mainly of CD3^+^ T lymphocytes predominated in several encephalic regions of the dogs from all the three infected groups, with no difference among them (*P* = 0.0004), whereas CD79α^+^ B lymphocytes were detected in very small intensity and presented no difference among groups (*P* = 0.5313). Furthermore, no association among diseases was detected at the serological enquire.

**Conclusions:**

We demonstrate that the peripheral infection by *L. chagasi per se* can promote the influx of lymphocytes within the nervous milieu as occurs during *Toxoplasma* and *Neospora* infections, and the concomitant seropositivity against these pathogens does not exacerbate the inflammatory brain lesions. Therefore, these findings give additional support that the brain should be included in the list of organs affected by visceral leishmaniasis and that even asymptomatic infected dogs may develop brain lesions.

## Background

Visceral leishmaniasis (VL) is a vector-borne disease caused by the intracellular protozoan *Leishmania chagasi* (Syn = *L. infantum*) and transmitted by phlebotomine sand flies. VL is a disease with great variability of clinical manifestations, and in dogs it ranges from absence of symptoms to generalized disorders that may result in the host death, such as fever, anemia, cachexia, skin lesions, lymphadenopathy, renal alterations and ocular lesions [1-3]. The clinical manifestations vary according to the host immune response and to the parasite pathogenicity, which differs among species [2]. Chronically infected dogs may develop neurological disorders, however, there are few reports that characterize the lesions and make clear the pathogenesis of the canine cerebral leishmaniasis (CCL) [4-7]. Toxoplasmosis is known by causing pulmonary and digestive disorders, retinal lesions and neuropathies [8-10], and canine neosporosis is an important cause of neurological disorders [11,12].

Canine VL is considered an immune-mediated disease due to the parasite ability to modify the host immune system. During the disease, infected cells from the mononuclear phagocyte system act as antigen presenting cells (APCs), stimulating CD4^+^ T helper (Th) lymphocytes. Th1 response, mediated by macrophages and pro-inflammatory cytokines such as TNF-α, IL-2 and IFN-γ is related to the host ability to control the infection. However, as an evasion strategy, components of the parasite preferentially activate Th2 lymphocytes and when the Th2 response predominates, the production of anti-inflammatory cytokines such as IL-4, IL-10 and TGF-β occurs, resulting in B lymphocytes proliferation and subsequent production of antibodies, mainly IgG, which are not effective against the parasite [2,13-15].

The involvement of the host immune system due to the *Leishmania* infection [16] may be related to the infection by other pathogens. In humans, the association between VL and AIDS is becoming common in VL endemic areas and HIV/*Leishmania* co-infection is considered an emerging disease [17,18]. Concomitant incidence of VL, neosporosis and toxoplasmosis in dogs have already been described in the region of the municipality of Araçatuba, São Paulo State, Brazil, an endemic area for VL [19]. Toxoplasmosis is an opportunistic disease related to immunosuppressive status [9,10,19,20] and association between canine VL and neosporosis has also been detected [19,21,22]. Recently, Lindsay et al. [23] confirmed the role of dogs as the definitive host of *N. caninum*, and even if dogs are not the definitive host of *T. gondii*, they may collaborate with mechanical dissemination of toxoplasmosis [9].

VL may be associated to an immunosuppressive state [13,14] and the contact between the host with the opportunist pathogens *Toxoplasma gondii* and *Neospora caninum* possibly will contribute to the occurrence of lesions in the central nervous system (CNS). Recently, we demonstrated that dogs with VL presented encephalic lesions compatible with encephalitis or at least, that the nervous milieu presented a pro-inflammatory status [5,24,25]. Hence, the aim of this study was to compare the lymphocytes population (T and B cells) in the brains of seropositive dogs to *L. chagasi* with those observed in dogs with seropositivity to *T. gondii* and *N. caninum*.

## Methods

### Animals

Eighty-one mixed-breed, male and female, adult dogs, which were referred to the Veterinary Teaching Hospital at UNESP - São Paulo State University, and to the local Zoonosis Control Center, between August/2006 and June/2009, were used in this study. The municipality of Araçatuba is an endemic area for VL with a seroprevalence of canine VL of 12% [26]. Blood samples were collected before the dogs were euthanized, in compliance with the State law, with an overdose of pentobarbital and potassium chloride. Necropsies were performed immediately after euthanasia, and gross lesions were recorded. None of the animals presented history of neurological symptoms.

### Parasitological and serological analysis

The diagnosis of VL was achieved by serological and parasitological examinations. Serological diagnosis was done using ELISA tests as described by Lima et al. [27] with the lower limit of positivity (cut-off) considered as 0.270 (optical density). Parasitological diagnosis of VL was achieved by finding typical amastigotes forms of *Leishmania* in cytological examinations of tissue smears from the popliteal lymph node.

The serological diagnosis of toxoplasmosis and neosporosis was performed using indirect immunofluorescence assays as described by Varandas et al. [28], being considered seropositives those dogs presenting antibody titer equal to or greater than 1:16 and 1:50, respectively.

### Experimental design

The dogs were divided into four experimental groups: (1) the LTN group was composed of 24 dogs with concurrent seropositivity to VL, toxoplasmosis and/or to neosporosis; (2) the L group was constituted by 31 dogs with seropositivity only to VL; (3) the TN group was composed of 16 dogs with negative diagnosis to VL and with seropositivity to toxoplasmosis and/or to neosporosis; and (4) the C group, constituted by 10 control healthy dogs with negative diagnosis for VL, toxoplasmosis and neosporosis, which death have not been related to CNS injury and which were referred by the owners to the Animal Pathology Service of UNESP - São Paulo State University in Araçatuba (Table 1). Age and gender were not taken into account in the analyses, since Gennari et al. [19] detected no association between gender or age and the prevalence of toxoplasmosis, neosporosis or visceral leishmaniasis.

**Table 1:** Gender and age range of the dogs included in this study, according to the experimental groups

**Group**		**LTN**		**L**		**TN**		**C**		**Total**	
		**n**	**%**	**n**	**%**	**n**	**%**	**n**	**%**	**n**	**%**
Gender	Male	15	62.5	16	51.6	7	43.8	7	70.0	45	55.6
	Female	9	37.5	15	48.4	9	56.3	3	30.0	36	44.4
	**Total**	24	100.0	31	100.0	16	100.0	10	100.0	81	100.0
Age range	0-1	2	8.3	7	22.6	1	6.3	2	20.0	12	14.8
(years)	1-2	3	12.5	9	29.0	4	25.0	2	20.0	18	22.2
	2-7	19	79.2	15	48.4	11	68.8	5	50.0	50	61.7
	>7	0	0.0	0	0.0	0	0.0	1	10.0	1	1.2
	**Total**	24	100.0	31	100.0	16	100.0	10	100.0	81	100.0

*LTN* Dogs with seropositivity to visceral leishmaniasis, toxoplasmosis and neosporosis, *L* Dogs with seropositivity only to visceral leishmaniasis, *TN* Dogs with seropositivity only to toxoplasmosis and/or neosporosis, *C* Control healthy dogs.

### Histopathology and immunohistochemistry (IHC)

The brain was sagitally sliced after removal and fixed in 10% neutral buffered formalin. Tissue samples were paraffin-embedded, sectioned (4 μm) and stained with haematoxylin & eosin (HE) using routine protocols. The following areas were evaluated for lesions: ventricular choroid plexi, ependyma and sub-ependymal area, leptomeninges in the temporal and piriform cortex and in the thalamus. The histopathological examination was done to verify the presence of inflammatory cells particularly in the perivascular, ventricular and sub-meningeal spaces.

IHC stains were performed using standard streptavidin-biotin peroxidase (HRP) immunostaining with polyclonal rabbit anti-human CD3 antibody (Dako A0452, Carpinteria, CA, USA) to detect CD3^+^ T lymphocytes, and the monoclonal mouse anti-human CD79αcy antibody (Dako M7051, Carpinteria, CA, USA) to identify CD79α^+^ B lymphocytes. Slides were deparaffinised in xylene and hydrated through graded alcohols. Antigen retrieval was achieved by steam heating in Tris-EDTA buffer plus 0.05% (v/v) Tween-20, pH 9.0, for 30 minutes. To inhibit endogenous peroxidase, slides were incubated with 2% (v/v) hydrogen peroxide 30 vol. diluted in 50% (v/v) methanol and deionized water for 30 minutes, and nonspecific binding was blocked with 3% (w/v) non-fat dry milk in phosphate-buffered saline (PBS) pH 7.2 for 30 minutes. Primary antibodies against CD3 (1:100), and CD79α (1:50) were applied for 18–22 hours at 4°C in a humidified chamber. Slides were washed in PBS, incubated with a biotinylated secondary antibody and streptavidin-HRP complex (Dako K0690 LSAB^+^ Kit, Carpinteria, CA, USA), then the reaction was developed with 3,3′-diaminobenzidine (Dako K3468, Carpinteria, CA, USA) and counterstained with Harris’s haematoxylin, dehydrated, cleared with xylene, and mounted with cover slips. For internal IHC control, sections of canine lymph node were used as positive control, and for negative control the primary antibody incubation was skipped in some slides. Tissue sections were examined by light microscopy. The presence of immunostained cells was analysed colorimetrically, quantifying the percentage of the immunostained area in relation to the total area, using the software Image-Pro Plus 6.1 (Media Cybernetics, Bethesda, MA, USA) according to the guidelines described by Melo and Machado [24]. Twenty images representative of twenty microscopic fields (90,506.04 μm^2^ per field, at the magnification of 40×) were obtained in four pre-defined encephalic areas: (1) leptomeninges in the temporal and piriform cortex; (2) ependyma and sub-ependymal area in the hippocampus; (3) leptomeninges in the thalamus; and (4) choroid plexus in the lateral ventricle, in a total area of 1,810,120.7 μm^2^. Examinations were done “blindly”, without knowledge of the experimental groups.

### Statistical analysis

The differences between groups were determined by the Kruskal-Wallis test followed by Dunn’s multiple comparison test. Association between the diseases was analysed using the Fisher’s exact test, and data were submitted to Spearman correlation test. A value of *P* < 0.05 was considered statistically significant. Data are expressed as the median (minimum - maximum), and all statistical analyses were performed using Prism 5 software (GraphPad, La Jolla, CA, USA).

### Ethics

This study was approved by the Institutional Ethics and Animal Welfare Committee (CEEA – Comissão de Ética e Experimentação Animal, UNESP – São Paulo State University, process number 05/06).

## Results

### Serological evaluation

No association between VL and the presence of anti-*N. caninum* antibodies (Fisher’s exact test: *P* = 0.8029) was detected, as well as between VL and the presence of anti-*T. gondii* antibodies (Fisher’s exact test: *P* = 0.1431). Table 2 illustrates the results of the serological enquire of the dogs according to the four experimental groups. Individual data from each animal may be found in Additional file 1: Table S1.

**Table 2:** **Occurrence of antibodies anti-*Toxoplasma gondii *and anti-*Neospora caninum *in dogs according to the positive or negative serology to visceral leishmaniasis**

**Group**	**Condition**	**Dogs**	
		***n***	**%**
LTN	*L. chagasi* positive, *T. gondii* positive and *N. caninum* positive	12	14.8
LTN	*L. chagasi* positive, *T. gondii* positive and *N. caninum* negative	4	4.9
LTN	*L. chagasi* positive, *T. gondii* negative and *N. caninum* positive	8	9.9
L	*L. chagasi* positive, *T. gondii* negative and *N. caninum* negative	31	38.3
	**Subtotal**	55	67.9
TN	*L. chagasi* negative, *T. gondii* positive and *N. caninum* positive	4	4.9
TN	*L. chagasi* negative, *T. gondii* positive and *N. caninum* negative	8	9.9
TN	*L. chagasi* negative, *T. gondii* negative and *N. caninum* positive	4	4.9
C	*L. chagasi* negative, *T. gondii* negative and *N. caninum* negative	10	12.4
	**Subtotal**	26	32.1
	**TOTAL**	81	100.0

### Necroscopic examination

The gross lesions found in the necroscopic examination are listed in Table 3. The main alteration observed in the dogs from the L group consisted by lymphadenopathy, skin lesions (ulcerations, alopecia, hyperpigmentation, seborrhoea, ear pinna ulcer), splenomegaly and weight loss/cachexia. Animals from TN group presented with skin lesions (alopecia, seborrhoea) as the major alteration, and few dogs showed lymphadenopathy, weight loss and splenomegaly, as well as other lesions, such as oral ulceration, hepatic lesions and hepatomegaly. In the dogs from the LTN group, it was observed the same alterations found in L and TN group, and further findings, such as eye lesions (blepharitis, purulent keratoconjunctivitis) and evident splenic white pulp. In all experimental groups, a high amount of dogs presented no macroscopic alterations and consequently they were considered asymptomatic. All alterations observed in the dogs were consistent with VL lesions and no other characteristic alteration of toxoplasmosis or neosporosis were detected.

**Table 3:** Gross lesions found in the necroscopic examination of the dogs, according to the experimental groups

**Alteration **^**1**^	**LTN group**		**L group**		**TN group**		**C group**	
	**(*n*** **= 24)**		**(*n*** **= 31)**		**(*n*** **= 16)**		**(*n*** **= 10)**	
	***n***	**%**	***n***	**%**	***n***	**%**	***n***	**%**
Evident splenic white pulp	2	8.3	1	3.2	-	-	-	-
Eye lesion	3	12.5	-	-	-	-	-	-
Lymphadenopathy	10	41.7	13	41.9	2	12.5	-	-
Onycogryphosis	1	4.2	1	3.2	-	-	-	-
Skin lesion	11	45.8	8	25.8	6	37.5	-	-
Splenomegaly	2	8.3	3	9.7	2	12.5	-	-
Weight loss	2	8.3	3	9.7	2	12.5	-	-
Other	-	-	-	-	3	18.8	-	-
No alteration	5	20.8	13	41.9	9	56.3	10	100.0

^1^One animal could have presented more than one alteration (*n* = 81).

*LTN* Dogs with seropositivity to visceral leishmaniasis, toxoplasmosis and neosporosis, *L* Dogs with seropositivity only to visceral leishmaniasis, *TN* Dogs with seropositivity only to toxoplasmosis and/or neosporosis, *C* Control healthy dogs.

### Histopathological findings

Remarkable inflammatory lesions were found in the nervous tissue of the dogs from all the infected groups. Inflammatory lesions, characterised by mononuclear cells, focal or coalescent multifocal accumulation, ranged in intensity from mild to severe. The lesions were composed mainly by lymphocytic cells, and they were evidenced in several encephalic regions, principally at the choroid plexus, ependyma and sub-ependymal area, leptomeninges and sub-meningeal areas, as well as in the perivascular spaces in the brain parenchyma (Table 4). Minimal or no alterations were found in the dogs from the C group, and when some alteration was identified, it was constituted by blood vessel walls hyalinization. None of the dogs presented typical amastigotes forms of *Leishmania* and either characteristic cysts of *Toxoplasma* or *Neospora* in the nervous tissue.

**Table 4:** Histopathological alterations observed in the brains of the dogs, according to the experimental groups

**Alteration **^**1**^	**LTN group**		**L group**		**TN group**		**C group**	
	**(*n*** **= 24)**		**(*n*** **= 31)**		**(*n*** **= 16)**		**(*n*** **= 10)**	
	***n***	**%**	***n***	**%**	***n***	**%**	***n***	**%**
Blood vessel walls hyalinization (choroid plexus)	3	12.5	9	29.0	1	6.3	3	30.0
Blood vessel walls hyalinization (leptomeninges)	4	16.7	4	12.9	1	6.3	1	10.0
Ependymal rosettes	2	8.3	2	6.5	-	-	-	-
Inflammatory lesions ^2^	18	75.0	17	54.8	10	62.5	-	-
Microhemorrhages	3	12.5	-	-	1	6.3	-	-
No alteration	6	25.0	7	22.6	5	31.3	6	60.0

^1^One animal could have presented more than one alteration (*n* = 81).

^2^Inflammatory lesions, predominantly mononuclear cell infiltrates, at the choroid plexus, ependyma, leptomeninges and around parenchymal blood vessels (perivascular cuffs).

### Lymphocytes in the nervous tissue

**Figure 1:**
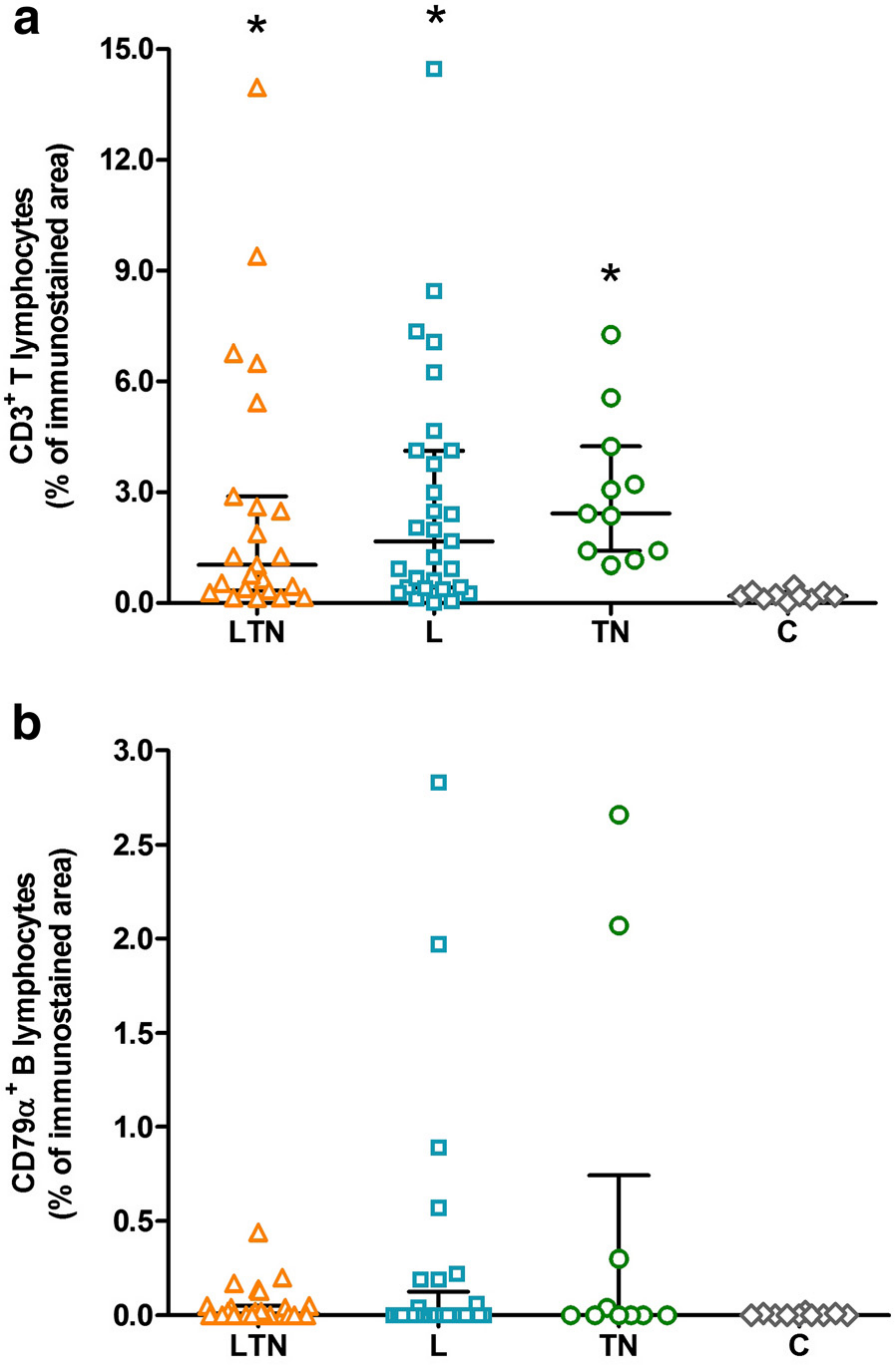
**Scatter plots showing the presence of T and B lymphocytes in the nervous tissue of the dogs. a**: CD3+ T lymphocytes. **b**: CD79α + B lymphocytes. Data are expressed as the percentage of immunostained area in relation to the total area, analysed colorimetrically by the software Image Pro-Plus 6.1. Horizontal lines indicate median (estimator of position measure) and the interquartile range (estimator of dispersion measure). LTN: dogs with seropositivity to visceral leishmaniasis, toxoplasmosis and neosporosis. L: dogs with seropositivity only for visceral leishmaniasis. TN: dogs with seropositive diagnosis only for toxoplasmosis and/or neosporosis. C: control healthy dogs. (* indicates P < 0.05).

CD3^+^ T lymphocytes predominated in the infected groups, when compared to control group (Kruskal-Wallis test: *P* = 0.0004). The LTN group presented, as the median value, 1.02% (0.13 - 13.96) of CD3 immunostained area, 1.66% (0.00 - 14.46) in the L group, and 2.42% (1.02 - 7.26) in the TN group, with no difference among them. The C group presented only 0.19% (0.01 - 0.47) of CD3 immunostained area (Figure 1a). Regarding CD79α^+^ B lymphocytes (Figure 1b), the LTN group presented 0.01% (0.00 - 0.44) of CD79α immunostained area, the L group 0.00% (0.00 - 2.83), the TN group 0.00% (0.00 - 2.66), and the C group 0.00% (0.00 - 0.02), with no significative difference among them (Kruskal-Wallis test: *P* = 0.5313).

**Figure 2:**
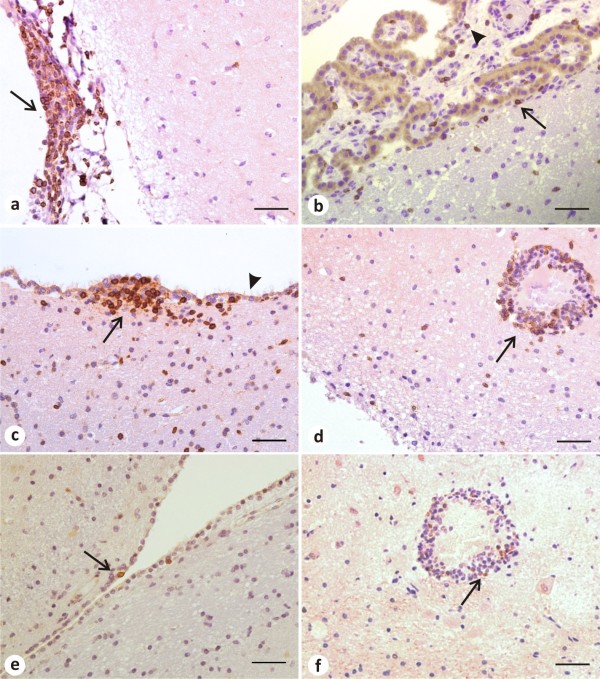
**Representative photomicrographies of immunohistochemical detection of CD3**^**+ **^**and CD79α**^**+ **^**lymphocytes within the nervous tissue of dogs (LTN group). a**: CD3^+^ T lymphocytes in the leptomeninges (*arrow*) from the temporal cortex as the major component of the inflammatory infiltrate. **b**: Disperse CD3^+^ T lymphocytes in the choroid plexus (*arrowhead*) and in the sub-ependymal area (*arrow*). **c**: Inflammatory infiltrate composed of CD3^+^ T lymphocytes (*arrow*) in the lateral ventricular ependyma. Note that the immunostained cells are just under the ciliated cells monolayer (*arrowhead*) that forms the ependyma. **d**: Perivascular cuff constituted mainly by CD3^+^ T lymphocytes (*arrow*) in the thalamus. **e**: Presence of two CD79α^+^ B cells (*arrow*) in the subependymal area. **f**: Few CD79α^+^ B cells (*arrow*) in the same perivascular cuff in the thalamus shown in **d**. Streptavidin–biotin peroxidase complex method. Scale bar = 50 μm.

CD3^+^ T lymphocytes were primarily distinguished as the major component of the inflammatory infiltrate around parenchymal blood vessels, in the ependymal zone, in the leptomeninges, as well as in the choroid plexus (Figure 2a-d). CD79α^+^ B lymphocytes, even if in small number, were detected basically in the leptomeninges and in the choroid plexus (Figure 2e-f). Evaluation of inflammatory cells presence according specific areas of the nervous tissue (choroid plexus, ependyma and leptomeninges) did not result in statistical relevance for CD3^+^ T lymphocytes (Kruskal-Wallis test: *P* = 0.8043) and neither for CD79α^+^ B lymphocytes (Kruskal-Wallis test: *P* = 0.0714). The correlation between antibody titers and cellularity was also tested. Correlation between *T. gondii*-antibody titers and CD3^+^ T lymphocytes was not significant (Spearman test: P = 0.7970), and neither between *N. caninum*-antibody titers and CD3^+^ T lymphocytes (Spearman test: P = 0.5857).

## Discussion

The data presented herein provide a picture of the lymphocyte population within the brain of dogs with seropositivity to visceral leishmaniasis, toxoplasmosis and neosporosis. Although *L. chagasi*, *T. gondii* and *N. caninum* are important pathogens, a high amount of the dogs presented no alterations at the necroscopic examination and consequently they were considered asymptomatic. An important epidemiological characteristic of VL is that infected dogs may remain asymptomatic for a long period and may never develop the clinical disease [2,14]. However, the presence or absence of clinical symptoms did not influence the histopathological lesions in the nervous tissue of naturally infected dogs [25]. None of the dogs presented signs of neurological disorder and no characteristic alterations of toxoplasmosis or neosporosis was observed, except for lymphadenopathy, which was the only symptom observed in the dogs that have been experimentally infected by different strains of. *T. gondii *[8].

Clinical neosporosis in adult dogs is generally related to the reactivation of a previous infection due to an immunosuppressive status [29], and the occurrence of clinical signs is associated with antibody titer greater than 1:800, while antibody titers equal to or greater than 1:50 only indicate exposure to the parasite [28]. Regarding toxoplasmosis, during experimental infection, some dogs keep asymptomatic, few dogs develop clinical signs and deaths are rarely seen [8].

Even if association among these diseases were not detected, 43.6% (24/55) of the dogs with VL presented antibody titers for *T. gondii* and/or *N. caninum*, and on the other hand, 61.5% (16/26) of the dogs diagnosed without VL presented antibody titers for *T. gondii* and/or *N. caninum*. The presence of positive antibody titer for toxoplasmosis and/or neosporosis in animals with VL did not influence the amount of CD3^+^ cells within the brain, as occurred in the dogs infected only by *L. chagasi* or in seropositive dogs only for *T. gondii* and/or *N. caninum*. All of these three conditions rendered favourable the accumulation of CD3^+^ T lymphocytes in the nervous milieu, since there was evident difference when compared to control group. Further, the great variability observed among the number of CD3^+^ T lymphocytes detected in the dogs within the same group is likely to be due to the natural character of the infection. Regarding the CD79α^+^ B lymphocytes, the presence of these cells was not significant in none of the above mentioned conditions, as the CD79α immunostaining was minimal and rather equivalent among groups. Although these cells play a role in the peripheral immune response against VL, they seem not to take part in the response within the brain.

The concomitance of VL, toxoplasmosis and neosporosis might be a propitious condition to the occurrence of neurological disorders. Alone, VL may promote an influx of a high amount of T lymphocytes, few phagocytic and B cells in the nervous tissue, primarily in the choroid plexus and leptomeninges, as well as high index of astroglial and microglial activation [24,25]. Further, the severity of the histopathological lesions and the presence of clinical neurological manifestation in dogs with VL seem not to be correlated [4]. Moreover, toxoplasmosis might promotes mononuclear meningitis as well as mononuclear perivascular cuffs [8] and in mice experimentally infected by *N. caninum*, the main encephalic alteration was encephalitis, glial nodules, malacia and perivascular cuffs [11]. Several of these histopathological alterations were detected in the brains of dogs included in this study, with inflammatory infiltrates being the most prevalent.

The activation of a systemic immune response promotes the peripheral activation of lymphocytes and their migration across the blood–brain-barrier (BBB) into the nervous environment and brain parenchyma, as previously described by some researchers [30-35]. BBB is extremely susceptible to the action of cytokines, which might activate the endothelial cells and increase the permeability of the barrier [36].

The anti-*Leishmania* immune response also includes the production of several cytokines [2,37], which might modulate the BBB selective filtration and allow the passage of inflammatory mediators and immunoglobulins [38]. Receptors for the Fc portion of the immunoglobulins are present in immune cells (mast cells, lymphocytes, macrophages) and also in neurons and glial cells. The activation of these receptors starts a succession of biological processes including phagocytosis, degranulation and cytokine genes activation [39,40]. High titers of anti-*Leishmania* antibodies in the CSF have already been described in dogs with natural VL [4,25,41,42], and, therefore, the presence of circulating antibodies within the CNS may contribute to the beginning of the inflammatory process, as previously described in immune-mediated and degenerative diseases of CNS [43,44].

## Conclusion

In dogs, VL can be associated with an influx of CD3^+^ T lymphocytes in the brain similarly as occurs during toxoplasmosis and neosporosis, and the concomitant presence of serum antibodies against *Leishmania*, *Toxoplasma* and *Neospora* does not exacerbate the inflammatory brain lesions. In animals presenting with neurological signs, the use of serological tests is an important tool to diagnose toxoplasmosis or neosporosis, and the encephalitis caused by these agents presents a well described pattern [8,9,11,12,28]. Nevertheless, this and other studies [4,24,25,42,45] demonstrated that the peripheral infection by *L. chagasi per se* can promote the influx of lymphocytes and other inflammatory changes within the nervous milieu. Therefore, these findings give additional support that the brain should be included in the list of organs affected by visceral leishmaniasis and that even asymptomatic infected dogs may develop brain lesions. Finally, VL has to be considered as differential diagnosis for inflammatory lesions in the brain.

## Competing interests

None of the authors of this paper has a financial or personal relationship with other people or organizations that could inappropriately influence or bias the content of the paper.

## Authors’ contributions

KPS participated in the design of the study, carried out the immunohistochemical reactions, and helped to draft the manuscript. GDM participated in the computer-assisted image analysis, performed the statistical analysis, and helped to draft the manuscript. GFM conceived of the study, participated in its design and coordination, and helped to draft the manuscript. All authors read and approved the final manuscript.

## Supplementary Material

Additional file 1: Table S1 Individual data from each dog included in the experimental groups. Click here for file
