# Supplementary material for: T and B lymphocytes in the brains of dogs with concomitant seropositivity to three pathogenic protozoans: Leishmania chagasi, Toxoplasma gondii and Neospora caninum
Source: BMC Res Notes. 2013 Jun 8;6:226. doi: 10.1186/1756-0500-6-226 (PMC3701587; doi:10.1186/1756-0500-6-226)
Supplement: Additional file 1: Table S1 — Individual data from each dog included in the experimental groups. [file 1756-0500-6-226-S1.doc]

**Additional file 1.**

**Table S1.** Individual data from each dog included in the experimental groups.

| **ID#** | **Sex** | **Breed** | **Age range (years)** | **Group¹** | **Parasitological²** | **Antibody titer³** | | |
| --- | --- | --- | --- | --- | --- | --- | --- | --- |
|  |  |  |  |  | ***Leishmania*** | ***Leishmania*** | ***Toxoplasma*** | ***Neospora*** |
| 10 | M | Poodle | 1-2 | LTN | + | 0.289 | 1:640 | 1:100 |
| 19 | F | Mongrel | 2-7 | LTN | - | 0.321 | 1:1200 | - |
| 21 | F | Poodle | 2-7 | LTN | - | 0.339 | 1:5120 | - |
| 22 | F | Mongrel | 2-7 | LTN | + | 0.401 | 1:640 | 1:200 |
| 29 | F | Mongrel | 1-2 | LTN | - | 0.294 | 1:640 | 1:200 |
| 34 | M | Mongrel | 2-7 | LTN | + | 0.430 | 1:2560 | 1:200 |
| 41 | F | Mongrel | 2-7 | LTN | - | 0.376 | 1:40 | 1:6400 |
| 43 | M | Mongrel | 2-7 | LTN | - | 0.301 | 1:1280 | 1:100 |
| 49 | M | Pit bull | 2-7 | LTN | + | 0.291 | 1:80 | 1:6400 |
| 61 | M | Mongrel | 1-2 | LTN | + | 0.335 | 1:80 | 1:400 |
| 67 | M | Pit bull | 2-7 | LTN | + | 0.324 | - | 1:800 |
| 70 | M | Mongrel | 2-7 | LTN | + | 0.335 | 1:40 | 1:800 |
| 77 | F | Mongrel | 2-7 | LTN | + | 0.378 | 1:1280 | 1:800 |
| 79 | M | Mongrel | 2-7 | LTN | - | 0.398 | 1:40 | 1:800 |
| 80 | M | Mongrel | 0-1 | LTN | - | 0.403 | 1:320 | - |
| 81 | M | Poodle | 2-7 | LTN | + | 0.392 | 1:320 | - |
| 83 | M | Poodle | 0-1 | LTN | + | 0.671 | 1:320 | 1:800 |
| 84 | M | Mongrel | 2-7 | LTN | + | 0.493 | 1:40 | 1:800 |
| 85 | M | Mongrel | 2-7 | LTN | + | 0.386 | 1:40 | 1:400 |
| 86 | M | Mongrel | 2-7 | LTN | + | 0.431 | 1:320 | 1:800 |
| 93 | F | Mongrel | 2-7 | LTN | + | 0.532 | 1:160 | 1:800 |
| 94 | F | Mongrel | 2-7 | LTN | + | 0.480 | 1:640 | 1:800 |
| 95 | F | Mongrel | 2-7 | LTN | + | 0.547 | 1:40 | 1:800 |
| 96 | M | Mongrel | 2-7 | LTN | + | 0.633 | 1:40 | 1:1600 |
| 11 | M | Pit bull | 0-1 | L | + | 0.412 | 1:8 | - |
| 12 | F | Pit bull | 0-1 | L | - | 0.623 | - | - |
| 13 | M | Mongrel | 1-2 | L | - | 0.387 | - | - |
| 14 | F | Rottweiler | 0-1 | L | + | 0.416 | - | - |
| 15 | F | Cocker spaniel | 1-2 | L | + | 0.284 | - | - |
| 16 | M | Teckel | 2-7 | L | + | 0.502 | - | - |
| 17 | F | Mongrel | 0-1 | L | + | 0.286 | - | - |
| 18 | F | Teckel | 1-2 | L | - | 0.593 | - | - |
| 20 | F | Pekingese | 2-7 | L | + | 0.450 | - | - |
| 24 | M | Mongrel | 1-2 | L | + | 0.279 | 1:2 | 1:40 |
| 25 | M | Teckel | 1-2 | L | + | 0.397 | - | - |
| 30 | M | Mongrel | 2-7 | L | + | 0.290 | 1:8 | - |
| 32 | F | Mongrel | 0-1 | L | + | 0.345 | - | - |
| 35 | M | Mongrel | 0-1 | L | - | 0.373 | - | 1:40 |
| 36 | M | Mongrel | 2-7 | L | + | 0.325 | 1:8 | - |
| 39 | F | Mongrel | 2-7 | L | + | 0.291 | - | - |
| 44 | M | Pit bull | 1-2 | L | + | 0.478 | - | 1:40 |
| 46 | M | Pit bull | 2-7 | L | + | 0.027 | - | 1:40 |
| 47 | M | Pit bull | 0-1 | L | + | 0.330 | - | 1:25 |
| 50 | F | Poodle | 2-7 | L | - | 0.500 | - | 1:40 |
| **ID#** | **Sex** | **Breed** | **Age range (years)** | **Group¹** | **Parasitological²** | **Antibody titer³** | | |
|  |  |  |  |  | ***Leishmania*** | ***Leishmania*** | ***Toxoplasma*** | ***Neospora*** |
| 52 | F | Labrador retriever | 2-7 | L | + | 0.388 | - | 1:40 |
| 54 | F | Mongrel | 2-7 | L | + | 0.292 | - | 1:40 |
| 55 | M | Pit bull | 1-2 | L | + | 0.303 | 1:2 | 1:40 |
| 56 | F | Mongrel | 2-7 | L | - | 0.462 | 1:4 | 1:40 |
| 60 | F | Teckel | 2-7 | L | + | 0.290 | 1:4 | 1:25 |
| 62 | F | Mongrel | 1-2 | L | + | 0.281 | - | - |
| 64 | F | Pinscher | 2-7 | L | - | 0.340 | - | 1:25 |
| 76 | M | Mongrel | 1-2 | L | + | 0.255 | 1:8 | - |
| 82 | M | Mongrel | 2-7 | L | - | 0.342 | 1:8 | - |
| 89 | M | Mongrel | 2-7 | L | - | 0.448 | - | - |
| 91 | M | Mongrel | 2-7 | L | - | 0.424 | - | - |
| 23 | F | Mongrel | 1-2 | TN | - | 0.137 | 1:640 | - |
| 26 | M | Mongrel | 1-2 | TN | - | 0.054 | 1:2560 | - |
| 37 | F | Mongrel | 2-7 | TN | - | 0.200 | 1:50 | - |
| 40 | M | Mongrel | 2-7 | TN | - | 0.213 | 1:2560 | - |
| 51 | M | Mongrel | 0-1 | TN | - | 0.145 | 1:40 | 1:50 |
| 57 | F | Mongrel | 2-7 | TN | - | 0.178 | 1:40 | 1:25 |
| 59 | F | Mongrel | 2-7 | TN | - | 0.014 | 1:640 | 1:25 |
| 63 | F | Mongrel | 1-2 | TN | - | 0.229 | 1:80 | - |
| 68 | M | Mongrel | 2-7 | TN | - | 0.168 | 1:40 | - |
| 72 | F | Mongrel | 2-7 | TN | - | 0.090 | 1:80 | 1:800 |
| 73 | M | Teckel | 1-2 | TN | - | 0.051 | - | 1:800 |
| 74 | F | Mongrel | 2-7 | TN | - | 0.165 | - | 1:800 |
| 75 | F | Mongrel | 2-7 | TN | - | 0.125 | - | 1:800 |
| 87 | M | Mongrel | 2-7 | TN | - | 0.252 | 1:640 | 1:200 |
| 88 | M | Mongrel | 2-7 | TN | - | 0.239 | - | 1:200 |
| 90 | F | Mongrel | 2-7 | TN | - | 0.155 | 1:80 | 1:80 |
| 27 | M | Mongrel | 2-7 | C | - | 0.044 | - | - |
| 31 | M | Mongrel | 2-7 | C | - | 0.005 | - | - |
| 38 | F | Pit bull | 0-1 | C | - | 0.251 | 1:2 | - |
| 42 | F | Mongrel | 0-1 | C | - | 0.030 | - | - |
| 48 | M | Rottweiler | > 7 | C | - | 0.198 | - | 1:40 |
| 58 | M | Mongrel | 2-7 | C | - | 0.130 | 1:8 | 1:25 |
| 65 | F | Mongrel | 1-2 | C | - | 0.229 | - | - |
| 66 | M | Mongrel | 1-2 | C | - | 0.256 | - | - |
| 71 | M | Mongrel | 2-7 | C | - | 0.187 | - | - |
| 92 | M | Mongrel | 2-7 | C | - | 0.211 | - | - |

¹ LTN: dogs with seropositivity to visceral leishmaniasis, toxoplasmosis and neosporosis. L: dogs with seropositivity only to visceral leishmaniasis. TN: dogs with seropositivity only to toxoplasmosis and/or neosporosis. C: control healthy dogs.

² Positive parasitological tests when finding typical amastigotes forms of *Leishmania* in cytological examinations of popliteal lymph node smears (-: negative; +: positive).

³ *Leishmania*: ELISA, cut-off 0.270. *Toxoplasma*: IFAT, cut-off 1:16. *Neospora*: IFAT, cut-off 1:50.
